# Earlier intra-arrest transnasal cooling may be beneficial

**DOI:** 10.1186/cc9723

**Published:** 2011-03-11

**Authors:** M Castren, P Nordberg, FS Taccone, JL Vincent, L Svensson, D Barbut

**Affiliations:** 1 Karolinska Institutet, Stockholm, Sweden; 2 Södersjukhuset, Stockholm, Sweden; 3 Erasme Hospital, Brussels, Belgium; 4 BeneChill, Inc., San Diego, CA, USA

## Introduction

Animal studies suggest a life-saving benefit for intra-arrest cooling. Transnasal evaporative cooling has sufficient heat transfer capacity for effective intra-arrest cooling and improves survival in swine. A 200-patient study showed transnasal cooling to be a safe and feasible method of intra-arrest cooling. The study also showed a solid trend to improved neurologically intact survival rates in those patients receiving intra-arrest transnasal cooling.

## Methods

To determine effects on neurologically intact survival at 90 days from the addition of intra-arrest transnasal cooling compared with hospital-based cooling alone, patients in witnessed cardiac arrest of any rhythm and with CPR ≤15 minutes after a 112 call were randomized to intra-arrest transnasal cooling versus standard ACLS care in two European EMS systems. Transnasal cooling (RhinoChill (RC); BeneChill Inc., San Diego, CA, USA) was initiated using a mixture of volatile coolant plus oxygen for rapid evaporative heat transfer. In treatment patients, cooling was initiated pre-ROSC, during ongoing CPR. Patients in both groups were cooled upon hospital arrival.

## Results

Forty-one patients have been included thus far. The median time from the 112 call for EMS to start CPR was 7 minutes and the time to initiate cooling was 17 minutes. ROSC was achieved in 8/19 (42%) of the RC group versus 8/22 (36%) of the control group. Site 1 initiated cooling at 11 minutes, and the ROSC rate at this site was 3/6 (50%) for RC and 1/9 (11%) for controls. EMS CPR was initiated at 5 minutes in RC versus 7 minutes in controls. Site 2 initiated cooling at 20 minutes, and the ROSC rate for this site was 5/13 (39%) for RC compared with 7/13 (54%) in the controls. EMS was initiated at 7 minutes in RC versus 9 minutes in controls.

## Conclusions

Initiating transnasal cooling extremely early during arrest may be superior to later intra-arrest initiation in relation to ROSC rates. The impact of this ultra-early cooling on outcome remains to be determined.

